# Polarized light detection in bumblebees varies with light intensity and is mediated by both the ocelli and compound eyes

**DOI:** 10.1098/rsbl.2024.0299

**Published:** 2024-09-25

**Authors:** Priscila Araújo, Gregor Belušič, Marko Ilić, James Foster, Keram Pfeiffer, Emily Baird

**Affiliations:** ^1^ Department of Zoology, Stockholm University, Stockholm 11418, Sweden; ^2^ Department of Biology, Biotechnical Faculty, University of Ljubljana, Večna pot 111, Ljubljana 1000, Slovenia; ^3^ Department of Neurobiology, University of Konstanz, Konstanz 78464, Germany; ^4^ Department of Behavioral Physiology and Sociobiology, Biocenter, University of Würzburg, Würzburg 97074, Germany

**Keywords:** dorsal rim area, dim light, vision, navigation, celestial cue

## Abstract

Like many insects, bumblebees use polarized light (PL) to orient and navigate. The celestial PL pattern is strongest when the sun is close to the horizon, during the dim light of dawn and dusk. In the dim light, the sensitivity of the compound eyes may not be sufficient for detecting PL or landmarks, and it has previously been hypothesized that bumblebees rely on PL from their more sensitive ocelli to navigate at dawn and dusk. Here, we tested this hypothesis using a combination of electrophysiological and behavioural tests. Specifically, we investigate whether bumblebee ocelli can detect PL and explore how the PL contribution from the ocelli and compound eyes is affected by light intensity. We find that bumblebee ocelli do indeed have PL sensitivity and that PL information can be used to guide behaviour in dim light. In bright light, however, both the compound eyes and ocelli are important for the detection of PL. Our results support the hypothesis that bumblebees use PL information from the ocelli at the low light levels that occur around dawn and dusk, and this may support their ability to forage during these periods.

## 1. Background

The sky provides insects with several cues for orientation and navigation. One of the most important of these cues is the pattern of polarized light (PL) that is generated in the sky when sunlight is scattered by the atmosphere and that moves with the sun throughout the day [1]. To detect PL, insects have specialized photosensitive structures (known as rhabdoms) that contain two untwisted photoreceptor cells that are located in the dorsal rim area (DRA) of the compound eyes [2–4]. Rhabdoms that satisfy the anatomical requirements for PL sensitivity have also been found in the dorsally located simple (camera-type) eyes called ocelli in some insects [5–9]. Electrophysiological investigations have also shown that photoreceptors in the ocelli of honeybees have PL sensitivity, although behavioural evidence suggests that they may not be used for PL-based navigation [10], a finding that is consistent with behavioural experiments in crickets [11]. The ability of ocelli to detect and use celestial compass cues has only been demonstrated behaviourally in ants, but whether they were using PL or other cues, such as the sun or the intensity or spectral gradients, remains unclear [12,13].

Wellington [14] hypothesized that the diurnal generalist bumblebee *Bombus occidentalis* uses PL information from its ocelli to guide flight and navigate during the dim light periods of dawn and dusk, when the DRA may not be sensitive enough to detect PL. This ability to prolong flight activity would be advantageous by allowing bumblebees to reduce competition for food by foraging when other diurnal bees cannot [14]. This hypothesis has been partly supported by anatomical evidence that photoreceptors in bumblebee ocelli are sensitive to PL [15], but direct evidence is lacking. Here, we aim to rigorously test the hypothesis that bumblebee ocelli detect PL and use it to guide behaviour using a combination of electrophysiological and behavioural experiments. We began by investigating if bumblebee ocelli respond to PL and characterizing the PL-sensitive responses recorded in the DRA and the main retina. We then performed behavioural experiments to investigate the relative contribution that the ocelli and DRA make in detecting PL at different light intensities. The combination of both approaches revealed that bumblebee ocelli are indeed sensitive to PL and that, in dim light, the ocelli provide the primary PL input, whereas in bright light, both the DRA and ocelli contribute to PL detection.

## 2. Methods

All experiments were performed on buff-tailed bumblebee workers (*Bombus terrestris*) acquired from Koppert Biological Systems (Berkel en Rodenrijs, The Netherlands). Colonies were fed ad libitum with sucrose solution (Koppert Natupol smart sucrose solution) and a mixture of sugar water and fresh-frozen organic pollen. Electrophysiological measurements were performed at the University of Ljubljana (Slovenia). Data were collected from seven workers from one colony. The behavioural experiments were performed at Stockholm University (Sweden) between December 2022 and August 2023. Colonies were housed at 24 ± 2°C under a 12 : 12 h light : dark cycle. During the training and testing phase, the sucrose solution was removed from the colony to motivate foraging behaviour. Workers were marked with tags for individual identification, and responses from a total of 436 workers from seven colonies were recorded.

### (a) Photoreceptor recordings

Dark-adapted workers were immobilized in a plastic pipette tip with beeswax and resin and mounted on a stage with goniometric and linear XYZ positioning capabilities that also carried a micromanipulator (Sensapex, Oulu, Finland). The animal and the micromanipulator moved in unison to facilitate stimulation from the optimal angle. For all recordings, an Ag/AgCl wire (50 μm in diameter) inserted into the head or thorax served as a reference electrode, and microelectrodes (Sutter, Novato, CA, USA) filled with 3 mol l^−1^ KCl solution (resistance 100−150 MΩ) were inserted into the retina. In the dorsal and ventral ocellar retinae, the microelectrodes were inserted through a small triangular hole in the head capsule located laterally from the ocellar lens and through a hole in the neural sheath around the brain. The location of the cells (i.e. dorsal or ventral) was determined according to the angle of stimulation: the visual axes in the ventral retina (dorsal visual field) were above 45°, while those in the dorsal retina (ventral visual field) were between 0° and 30°. For the DRA and main retina recordings, the microelectrodes were inserted through a small triangular hole in the cornea, ventral to the DRA. The signal was amplified using an SEC-10LX amplifier (npi electronic, Tamm, Germany), conditioned with a CyberAmp 320 (Axon Instruments, Union City, CA, USA) and digitized with a Micro1401 (CED, Cambridge, UK). Spectral stimulation was provided by an LED array ‘LED synth’ [16], and a xenon arc lamp (XBO, Cairn, UK) filtered with a monochromator (B&M, Limburg, Germany). The light sources were tuned to emit an equal number of photons at every wavelength (‘isoquantal’ mode). A UV transmissive polarization filter (OUV2500; Knight Optical, UK) was mounted in a motorized rotator (Qioptiq, Germany) and inserted into the stimulation beam to deliver PL at different angles. All cells were first stimulated with the LED synth for 2 s to determine their spectral sensitivity, after which their polarization sensitivity (PS) was measured at the peak wavelength of their spectral sensitivity. This was followed by measuring the intensity response function and a detailed spectral scan with a monochromator. The response amplitudes of single cells were transformed to sensitivities (S) by means of an intensity response function and a reverse Hill transformation [15]. Polarization sensitivity was calculated as the ratio between the sensitivity maximum and minimum, that is, PS = *S*_max_/*S*_min_ [17].

### (b) Behavioural experiments

**Figure 1.**
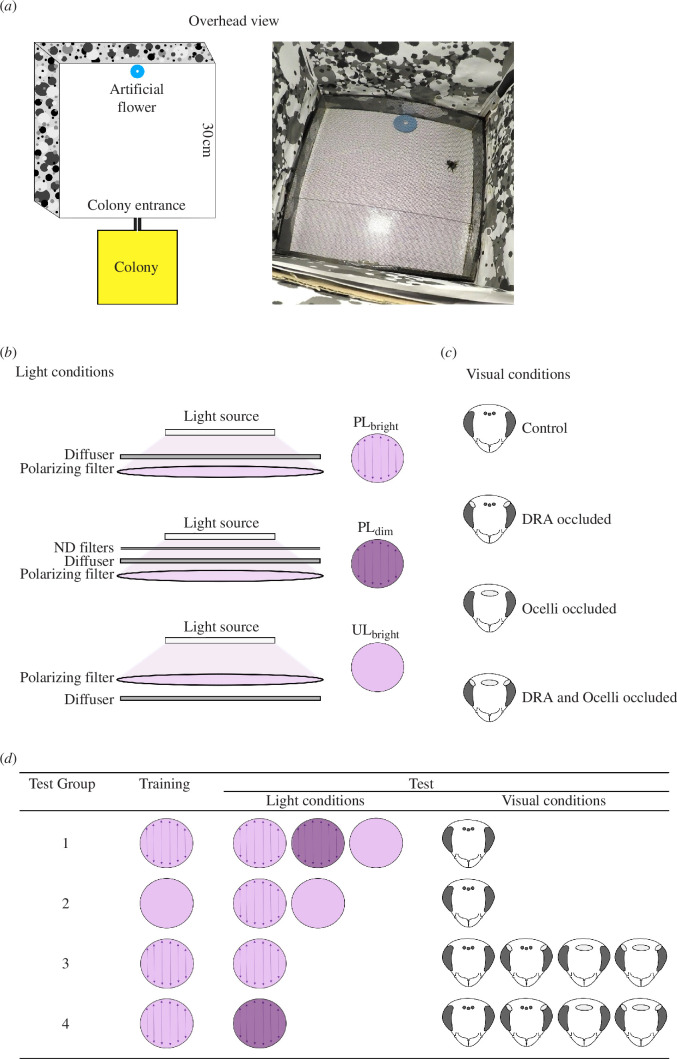
Overview of the experimental set-up and design. (*a*) Overhead view of the arena. A bumblebee colony (yellow box) was connected to the arena (white box) with tubes (black lines). Inside the arena, there was an artificial flower (blue circle) with a sugar solution reward. Right: a photo of a bee foraging inside the arena during the test phase (day 5). (*b*) The different light conditions used: PL_bright_, PL_dim_ and UL_bright_. ND filters = neutral density filters. (*c*) The different visual conditions used: control, DRA occluded, ocelli occluded and DRA and ocelli occluded. (*d*) Details of conditions for training and testing in each test group.

The behavioural experiments were conducted in an arena 30 cm × 30 cm × 35 cm (l × w × h) covered by a net placed at 3 cm height, so the bees in the arena could only walk (figure 1*a*). The colony was connected to the arena with two gated tubes. An artificial flower (a blue circle of laminated paper, 4.5 cm in diameter) was placed approximately 30 cm from the arena’s entrance. An Eppendorf tube containing 50% sugar solution was placed in the centre of the flower. The arena walls were covered with a grey-scale pattern [18] (figure 1*a*). A webcam was placed on one wall to observe activity in the arena. A light set-up consisting of a wide spectrum light (UV+, blue+, green+ and red+; Philips, LED Floodlight, IP65), a diffuser (an acrylic sheet that was polished such that it depolarized the light and ensured an even intensity pattern) and a polarizing filter (UV/VIS, Bolder Vision Optik), was placed 35 cm from the arena floor (figure 1*b*). Three configurations were used to produce different light conditions in the arena (figure 1*b*):

PL_bright_: the diffuser was placed between the light and the polarizing filter (figure 1*b*), making the light in the arena polarized. The absolute irradiance in the UV (320–360 nm), blue (405–445 nm) and green (505–545 nm) parts of the spectrum after the integration using the *sfsmisc* package [19] in R were 5.2, 47 and 249 μW cm^−2^, respectively.

PL_dim_: the same set-up as PL_bright_ was used, and to decrease the light intensity, two layers of a neutral density filter were placed above the diffuser (figure 1*b*). The absolute irradiance, after integration, in the UV, blue and green parts of the spectrum decreased to 1.2, 13 and 92 μW cm^−^², respectively. These values approximate the solar irradiance we measured when the elevation of the sun was 1° above the horizon. At this time, the absolute irradiance was 18, 29 and 27 μW cm^−^², respectively.

UL_bright_: the diffuser was placed under the polarizing filter (figure 1*b*) such that only unpolarized light (UL) reached the arena. The absolute irradiance was the same as for PL_bright_.

Each test group was separated into a training and a testing phase. In the training phase, bees had access to the arena from 08.00 to 16.00 over 4 days, during which time they could feed freely on the flower (which was refilled continuously) under either PL_bright_ or UL_bright_ light conditions (figure 1*b*), depending on the test group (details below).

After the training phase, depending on the test group, the ocelli and/or DRA of individual bees were occluded first by painting with one layer of black paint and then adding a second layer of white paint (Biltema touch-up pen, black matte and white 008), which allowed an observer to easily check if the paint was present. The visual conditions included a control with no occluded visual structures (i.e. all visual structures could provide PL, figure 1*c*), DRA occluded (i.e. PL information provided primarily by the ocelli, figure 1*c*), ocelli occluded (i.e. PL information provided primarily by the DRA, figure 1*c*) and DRA and ocelli occluded (i.e. PL information provided only by the non-DRA region of the compound eye, figure 1*c*).

Bees that experienced the training phase (which could be identified from their ID tags) were tested on day 5 by releasing them individually into the arena (by opening the tubes from the colony) and allowing them to search for the flower. The main goal of this experiment was to test whether bumblebees could detect PL in different light intensities and use different parts of the visual system to do so, not whether they could use PL for orientation specifically. As a result, the position of the artificial flower and the orientation of the polarization filter were kept in the same position throughout the training and testing phase. A floral visit (scored as 1) was recorded when the bee climbed onto the flower, extended its proboscis and fed. If this did not occur within 90 s, the test was scored as 0. Between each test, the arena was cleaned with 70% ethanol, and the flower was replaced with a clean one.

The different light and visual conditions were tested according to the test group below:

Test group 1 (figure 1*d*): bees with no visual structures occluded experienced PL_bright_ during the training phase and were tested with PL_bright_, PL_dim_ or UL_bright_. This allowed us to investigate if bees had associated floral visits with the presence of PL and if they were able to detect it in dim light.

Test group 2 (figure 1*d*): bees with no visual structures occluded experienced UL_bright_ during the training phase and were tested in UL_bright_ or PL_bright_. This allowed us to investigate if bees associated floral visits with a cue that was not PL.

Test group 3 (figure 1*d*): bees with different visual conditions experienced PL_bright_ during training and PL_bright_ during testing. This allowed us to investigate the contribution of the main retina, DRA and ocelli to detect PL in bright light.

Test group 4 (figure 1*d*): bees with different visual conditions experienced PL_bright_ during training and PL_dim_ during testing. This allowed us to investigate the contribution of the main retina, DRA and ocelli to detect PL in dim light.

### (c) Statistical analysis

Generalized linear mixed models and generalized linear models with a binomial distribution were used to analyse the data in each test group. All analyses and graphs were performed using R Cran Project v. 4 software [20] and the following packages: *lme4* [21], *afex* [22], *multcomp* [23] and *ggplot2* [24].

For test groups 1 and 2, we analysed the effects of different light conditions on the proportion of successful floral visits. Floral visit was the response variable (1 or 0), and light condition was the fixed effect (PL_bright_, PL_dim_ and UL_bright_). Bee and colony ID were used as random effects in the full model, but they were removed from the final model as both had singular fits. For test groups 3 and 4, we analysed the effects of visual structure occlusion on floral visits. Floral visit was the response variable (1 or 0), and occluded visual structure was the fixed effect (control, ocelli occluded, DRA occluded and DRA and ocelli occluded). Bee and colony ID were used as random effects in the full model in both tests. In test group 3, colony ID was removed from the final model as it had a singular fit. In test group 4, both were removed from the final model as both had a singular fit. If the fixed variable influenced the response variable, we performed planned comparisons between the treatments in all models.

## 3. Results and discussion

### (a) Spectral and polarized light sensitivity in bumblebees

**Figure 2.**
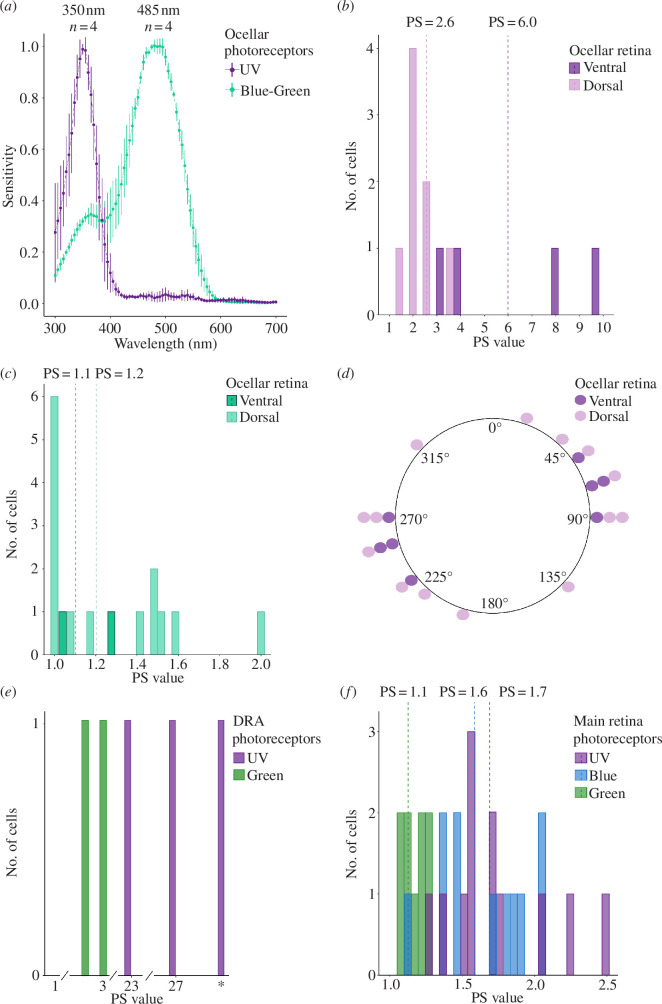
Intracellular photoreceptor recordings from bumblebee ocelli, DRA and main retina. (*a*) Spectral sensitivity of the ocelli. PS values of (*b*) UV and (*c*) blue-green cells in the ocelli. Dashed lines indicate the mean PS for the ventral (dark colour) and dorsal retina (light colour). (*d*) The circular histogram shows the UV cell’s angular sensitivity maxima of the ventral (dark colour) and dorsal (light colour) retina, which correspond, respectively, to the dorsal and ventral visual field. (*e*) PS values of UV and green cells in the DRA. The asterisk indicates a UV cell for which it was impossible to accurately calculate the PS due to negative responses at unpreferred polarizer orientations. (*f*) PS values of UV, blue and green cells in the main retina. Dashed lines indicate the PS means.

We could not distinguish any functional differences between cells from the median and lateral ocelli, so we merged the recordings from them for further analysis. Two types of spectrally distinct photoreceptors were detected, one with a peak at 350 nm (*n* = 4, figure 2*a*), here called a UV cell, and another with a main peak at 485 nm and a smaller peak at 380 nm, here called a blue-green cell (*n* = 4, figure 2*a*). Thus, like honeybees [9], bumblebees have two classes of photoreceptors in the lateral and median ocelli. The PS of UV cells was found to be moderate and high in the dorsal and ventral retinae, respectively (PSUV_dorsal_ ≈ 2.6 ± 0.6, *n* = 8 and PSUV_ventral_ ≈ 6.0 ± 3.2, figure 2*b*; receptor potentials and PS curve in eletronic supplementary material, figures S1 and S2, respectively). Blue-green cells in both retinae had lower PS (PSBG_dorsal_ ≈ 1.1 ± 0.3, *n* = 14 and PSBG_ventral_ ≈ 1.2 ± 0.1, *n* = 2, figure 2*c*; receptor potentials and PS curve in electronic supplementary material, figures S1 and S2, respectively). The UV cells in the dorsal retina varied in the angle of PL at which they were most sensitive, but the photoreceptors in the ventral retina were mostly sensitive to horizontal PL (figure 2*d*). Our results support the anatomical evidence that the ventral (i.e. sky-viewing) ocellar retina of bees detects PL [5,7]. Our data also suggest that if the dorsal retina indeed plays a role in flight stabilization by viewing the ventral visual field (as proposed by previous studies [5,7,9,25]), this may be achieved using PL in the UV part of the spectrum.

In the DRA, we discovered that UV cells had extremely high PS and that green cells had moderate PS (PSUV > 23, *n* = 3 and PSGreen ≈ 2.8 ± 0.1, *n* = 2, figure 2*e*; receptor potentials, PS curve and spectral sensitivity inelectronic supplementary materials, figures S1-S3, respectively). In the main retina of the compound eye, the UV and blue cells had low PS (PSUV ≈ 1.7 ± 0.3, *n* = 12 and PSBlue ≈ 1.6 ± 0.4, *n* = 11, figure 2*f*), while the green cells had negligible PS (PSGreen ≈ 1.1 ± 0.06, *n* = 9, figure 2*f*; spectral sensitivity in electronic supplementary material, figure S1). The recordings were made from cells located in the frontal and lateral regions of the eye. As the field of view in these regions corresponds mainly to lateral and frontal views [26] and is unlikely to receive visual input from the sky, our data suggest that, in the UV and blue parts of the spectrum, bumblebees have the substrate for non-celestial polarization vision, like some other arthropods [27]. In addition to being present in the sky, PL is also reflected from the surface of water, plants and insect cuticle [1]. If or how bees may use non-celestial PL remains unclear, but there is some evidence that it could be used in flower detection [28], and recently, PL patterns have been described in the wings of *Xylocopa latipes* bees, suggesting that it may mediate conspecific recognition [29].

### (b) Bumblebees detect polarized light at a range of light intensities

**Figure 3.**
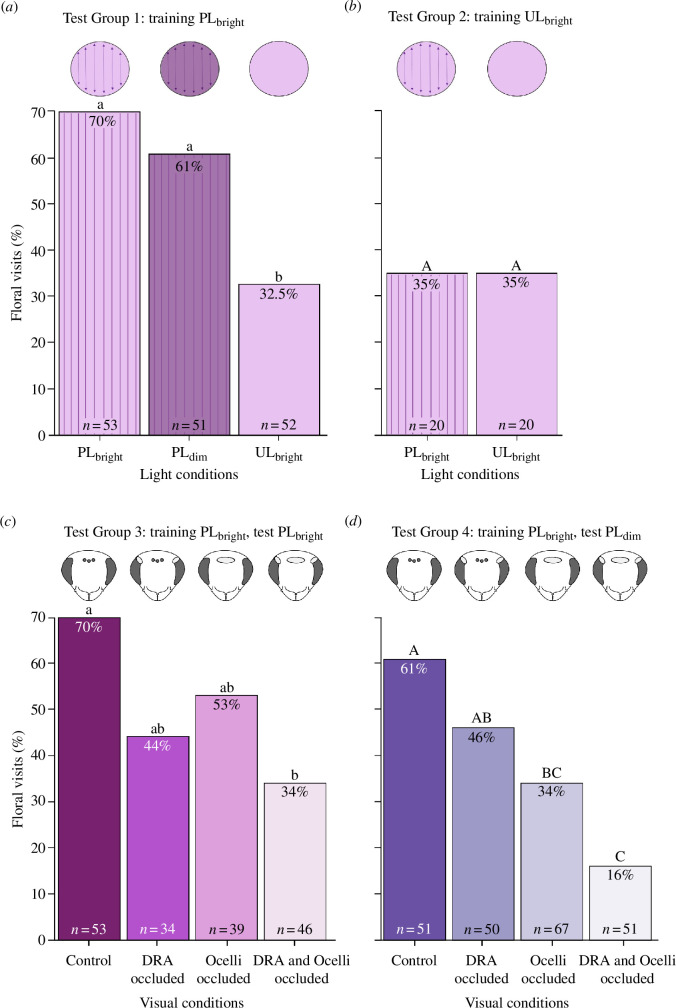
Floral visitation rates in different light intensities and in the presence or absence of PL after training in (*a*) PL_bright_ (test group 1) and (*b*) UL_bright_ (test group 2). Percentage of floral visitation in (*c*) PL_bright_ (test group 3) and (*d*) PL_dim_ (test group 4), after training in PL_bright_ with control (workers with ocelli, DRA and main retina available), DRA occluded (ocelli and main retina available), ocelli occluded (DRA and main retina available) and DRA and ocelli occluded (only main retina available).

When trained under PL_bright_ (test group 1, figure 1*d*), the proportion of bees that performed floral visits was affected by the light condition (figure 3*a*, table 1). Our results suggest that light intensity had no effect on the proportion of floral visits (approx. 65%; *z* = 0.96; *p* = 0.59, figure 3*a*). However, significantly fewer bees made floral visits under unpolarized light (UL_bright_) than under both bright and dim PL (PL_bright_: *z* = 3.7, *p* < 0.001; PL_dim_: *z* = 2.8, *p* = 0.01; figure 3*a*), suggesting that bees were more likely to visit the flower when PL was present, even in dim light. When trained under UL_bright_ (test group 2, figure 1*d*), only 35% of bees tested under UL_bright_ or PL_bright_ visited the flower (figure 3*b*), suggesting that bees trained with PL in test group 1 learned to associate PL, rather than any other cue, when foraging for the flower.

**Table 1.** Number of colonies, bees and the output of statistical models for test groups 1–4. A binomial distribution was used in all models. *n* = number of colonies and bees, *N* = number of trips.

test group	colony (*n*)	bee ID (*n*, *N*)	model	*χ* ^2^	d.f.	*p*‐value
1	3	141, 166	floral visit–light condition	16.0	2	<0.001
2	1	40, 40	—	—	—	—
3	7	164, 172	floral visit–eye treatment + (1|bee ID)	9.94	3	0.01
4	3	161, 229	floral visit–eye treatment	23.4	3	<0.001

### (c) Polarized light detection is mediated by the dorsal rim area and ocelli in bright light and by the ocelli in dim light

Floral visitation in PL_bright_ (test group 3, figure 1*d*) was influenced by the type of visual structure that was occluded (figure 3*c* and table 1). Bees with DRA or ocelli occluded had a similar percentage of floral visits to the control (figure 3*c* and table 2), suggesting that the DRA and ocelli play a significant role in detecting PL in bright conditions. This result is supported by our electrophysiological recordings, which show that both the DRA and the ocelli are PL-sensitive. Interestingly, only 34% of bees with both ocelli and DRA occluded visited the flower (figure 3*c*), which is close to the proportions recorded for bees with no visual structures occluded in the UL_bright_ condition of test groups 1 and 2. This similarity suggests that the DRA and the ocelli are the primary sources of PL information under these conditions and that PL detection was not mediated by the PL-sensitive cells in the main retina. In PL_dim_ (test group 4, figure 1*d*), the proportion of floral visits depended on the visual structure that was occluded (figure 3*d* and table 1) and was reduced in comparison to the control in all treatments, except for DRA occluded (*z* = 1.4, *p* = 0.25; figure 3*d* and table 2). As the ocelli would have been the only visual structure providing PL information, this result suggests that, in dim light, the ocelli contribute more to the detection of PL than the DRA. Taken together, the results from test groups 3 and 4 suggest that PL information from the DRA decreases in dim light while PL information from the ocelli is unaffected by light intensity.

**Table 2.** Results of the pairwise comparison for test groups 3 and 4. Bold text indicates significance at *p *< 0.05.

treatments	*z*-values	*p*‐value
test group 3—PL_bright_		
control bees versus DRA occluded	2.2	0.06
control bees versus ocelli occluded	1.5	0.25
control bees versus DRA and ocelli occluded	**3.1**	**0.004**
DRA occluded versus ocelli occluded	−0.8	0.99
DRA occluded versus DRA and ocelli occluded	0.8	0.61
ocelli occluded versus DRA and ocelli occluded	1.7	0.18
test group 4—PL_dim_		
control bees versus DRA occluded	1.5	0.27
control bees versus ocelli occluded	**2.8**	**0.01**
control bees versus DRA and ocelli occluded	**4.3**	**<0.001**
DRA occluded versus ocelli occluded	−1.2	1
DRA occluded versus DRA and ocelli occluded	**3.1**	**0.005**
ocelli occluded versus DRA and ocelli occluded	2.1	0.08

## 4. Conclusion

Our findings provide the first evidence that bumblebees can detect PL with their ocelli and DRA to guide foraging behaviour. Our results are consistent with the hypothesis that bumblebee ocelli support foraging at dusk and dawn when PL information is strong but light intensity approaches the limit of what the DRA can detect [14]. Because the proportion of bees that visited the flower with either the DRA or ocelli covered was consistently higher than when both the DRA and ocelli were covered but was consistently lower than bees that could use both structures, our data extend this hypothesis by indicating that PL information from the ocelli and DRA are combined to improve PL sensitivity. Overall, the results of this study provide a strong foundation for further experiments exploring how PL information from the DRA and ocelli could be used to guide insect foraging and navigation under natural conditions.

## Data Availability

The full dataset used to generate the figures and perform the statistical analyses presented is available in the electronic supplementary material [30].
